# Use of social media in food safety in Saudi Arabia—a preliminary study

**DOI:** 10.3934/publichealth.2021025

**Published:** 2021-03-29

**Authors:** Nisreen M Abdulsalam, Marwan A Bakarman

**Affiliations:** 1 Department of Food and Nutrition, Faculty of Human Sciences and design, King Abdul Aziz University, P.O. Box 42807, Jeddah 21551, Saudi Arabia; 2 Department of Family and Community Medicine, Rabigh Faculty of Medicine, King Abdul Aziz University, P.O. Box. 80205, Jeddah 21589, Saudi Arabia

**Keywords:** social media, food safety education, infection, food standards, public health

## Abstract

Over the past two decades, the rapid rise of social media has revolutionized the way we communicate and share information online. Social media platforms are not now only used extensively by individuals but also by businesses, governmental agencies, educational institutions, and many other organizations to deliver information to the public and, in return, collect information from that same audience. The preliminary study presented here offers valuable insights into how social media may be used to improve food safety standards. Today, food safety is still a major health challenge in the country, which occasionally faces unsafe food supply chains, an increased number of food borne outbreaks, and poor hygiene education. Social media may be used as a very valuable tool for people to access important information and more knowledge about food safety. The limited-scope survey presented here was conducted over the western part of Saudi Arabia and included 295 individuals of both genders, among various age groups. Participants responded to an online questionnaire about their use of social media to obtain information about food safety. Results showed that social media was indeed a major outlet for individuals to access information on food safety, with the top-ranked social media platforms being WhatsApp (*M* = 2.99) followed by Snapchat (*M* = 3.72), YouTube (*M* = 4.08), Instagram (*M* = 4.46), and Facebook (*M* = 4.81). Additionally, we found that the most trusted sources of information was the Saudi Food and Drug Authority (72.6%) and the Saudi Ministry of Health (55.4%). Participants most frequently sought epidemiological information (52.5%), quantitative risk estimates (23.1%), and information on the various types of foodborne infections (15.3%); they preferred the information to be in video format (67.5%), articles (57.6%), infographics (55.3%). Trustworthiness clearly emerged from the survey as an important consideration for individuals when accessing food safety information on social media.

## 1. Introduction

Food safety is a major challenge worldwide. Only last year, it was listed as one of the top three global issues by KRC Research in an online survey of 1,754 adults ages 18 to 65, with participants from the United States, United Kingdom, and China [1].

Just like many other countries, in particular developing countries, such as Saudi Arabia still faces many food safety issues, and it has become a major health concern for the country. According to the Saudi Ministry of Health (MOH) there were 2,191 hospital admissions linked to foodborne illnesses in 2018. In addition, there were 10.02 cases of amoebic dysentery per 100,000 of the population and 6.12 cases of Salmonella infection per 100,000 of the population that same year [2]. In 2015, a cross-sectional study among female individuals in Riyadh, Saudi Arabia, found that 45% followed unsafe food handling practices and lacked knowledge about food safety [3]. However, another cross-sectional study among Saudi university students performed two years later showed an eagerness to learn, with approximately 66% of female participants responding they were interested in learning more about food safety [4]. That same study also showed that male students were not aware of appropriate temperature controls for safely preparing and storing food.

Over the last few decades, new communication technologies have made information-sharing quicker, easier, and less expensive [5],[6]. Social media may offer a valuable option to help educate the Saudi public about food safety, in particular the young population. Indeed, Saudi Arabia, with a current total population of 34.54 million individuals, has 25 million individuals (72.38% of the total population) which are active social media users, with a majority of them being young people (75%). Saudi Arabia is now at the top position on the global social media scene [7]. The most frequently used social media platforms include (but are not limited to) Facebook, Twitter, Instagram, YouTube, and LinkedIn. [8]. In the United States, approximately 86% of adults above 18 years old use the internet, and 73% of those are registered with at least one social media platform [9]. This situation is similar in other developed countries [10]. In recent years, the use of social media over the internet has increased almost three times faster than the use of internet in general [11], [12]. The rapid spread of smart phones, and other digital mobile devices has further increased access to social media [13]. Unfortunately, social media is often used as a tool for disinformation, such as minimizing the risks of certain foods or exaggerating the health risks of others [14]. Such possibility of disinformation should always be taken into account when analyzing social media messages.

Public health organizations and companies cannot ignore the rapidly evolving means of access of information by consumers and individuals often seeking information directly from social media platforms [10]. The health care industry needs to recognize the importance of social media in order to better inform and educate communities [15], especially during major health crises such as the COVID-19 pandemic [16]–[18], with search engines often the starting point to explore relevant information [19]. Major food safety agencies, such as the U.S. Food and Drug Administration, the Saudi Food and Drug Authority, and the Saudi Ministry of Health, already use social media extensively [20].

The onset of the COVID-19 pandemic has brought additional challenges to the global food supply. It was hypothesized that humans might have contracted the virus by eating meat [21]. However, this hypothesis was eventually overturned. The World Health Organization has provided no evidence that the COVID-19 virus spreads through food or food packaging. In order to reassure the public, it is important to issue this statement in a timely manner and social media may suit this purpose perfectly.

The survey presented here offers preliminary data with respect to the use of social media in Saudi Arabia to access information about food safety. Although limited in scope, it does provide some directions for future investigations into the use of social media by the Saudi to get more knowledgeable about food safety and to improve the many challenges faced by the country due to insufficient food hygiene, and unsafe food practices.

## 2. Methodology

The survey was performed through the means of an online questionnaire among approximately 300 individuals from the western part of the country, both male and female among different age groups to decrease the risk of sampling biases, participants were recruited using multiple methods, including bulletin board posts, word of mouth, flyers, and announcements. Flyers played an essential role in diversifying the sample and reducing sampling biases because they were widely distributed. Places of distribution included hospital waiting rooms, restaurants, cafes, schools, university classrooms, and various other places. Each flyer included a description of the study and an online link to the survey. Anyone with a mobile device could enter the link printed on the flyer and take the online Arabic language questionnaire delivered through the LimeSurvey platform [22]. Prior to completion of the questionnaire, participants were asked to provide informed consent electronically. All data were analyzed using the SPSS Version 24 software. The focus of the analysis was on assessing the participants' perceptions of various social media platforms, such as Twitter and Facebook. Specifically, relative preference ranks for various social media platforms were requested. Additionally, perceptions of the platforms, such as trustworthiness, timeliness, security, or searchability, were assessed. Most analyses were descriptive in nature.

Preferred sources of information about food safety were compared across various demographic categories. All these analyses were bivariate and compared media preference independently across categories of marital status, age, region, gender, and education. In these comparisons, the dependent variable represented the preference score, while the independent variable represented categories within each demographic variable. For example, preference scores for the Internet were compared for males and females. The nonparametric Kruskal Wallis H-test and Mann Whitney U-test were used to make these comparisons.

### 2.1. Collection of data

**Design of the study:** The questionnaire for this study was developed based on an extensive review of the literature that discussed the use of social media for disseminating information on food safety. Sixteen questions were included, of these sixteen questions, six were about the demographic characteristics of the participants, and among the remaining ten questions, there was one free-response question, three Likert-type items, and six multiple-choice questions.

The questionnaire was developed through an iterative process that involved multiple rounds of reviews and corrections by five subject matter experts. In each round, experts read questionnaires and offered suggestions for improvement. After several iterations, a questionnaire was found satisfactory by the entire panel of experts and pilot tested. The test was performed over a group of eleven individuals recruited using a ‘Snowball sampling’ method [almost], also known as Chain Referral sampling method. Each participant was asked to comment on the clarity and logical adequacy of each question, and some of the suggestions identified by the participants were incorporated in the final version of the questionnaire.

Evaluation tools: Through this process, both the face and content validity of the questionnaire were established. The test-retest reliability was also investigated by asking the participants to answer all questions again. The reliability coefficients for the Likert-type items ranged between 0.67 and 0.81, and for the multiple-choice questions, the post-test matches were either 81.8% or 90.9%. Overall, these values suggest a reasonable degree of test-retest validity. Cronbach's alpha was not computed because such a computation is only appropriate for a group of items reflecting the same underlying construct. In this questionnaire, no such groups were present.

## 3. Results

### 3.1. Demographic profiles of the respondents

**Table 1.** Demographic characteristics of the participants (*n* = 295, Questions 1–6).

Demographic Characteristics	N	%	Demographic Characteristics	N	%
Marital status			Gender		
Married	78	26.4%	Female	213	72.2%
Divorced	10	3.4%	Male	82	27.8%
Separated	2	0.7%	Occupational status *		
Widowed	3	1.0%	Full-time employment	81	27.5%
Unmarried	202	68.5%	Part-time employment	75	25.4%
Age			Unemployed	10	3.4%
Under 18	40	13.6%	Self-employed	3	1.0%
19–29	152	51.5%	Homemaker	5	1.7%
30–39	60	20.3%	Student	167	56.6%
40–49	30	10.2%	Retired	7	2.4%
50 and over	13	4.4%	Other	8	2.7%
Region			Level of Education High school	23	7.8%
Middle	23	7.8%	Some college	140	47.5%
Northern	10	3.4%	Bachelor's degree	111	37.6%
Southern	33	11.2%	Master's degree	3	1.0%
Eastern	24	8.1%	Doctoral degree	5	1.7%
Western	205	69.5%	Professional degree	6	2.0%
			Other	7	2.4%

Note: * Percentages do not add up to 100% because multiple categories could be selected.

The survey included 295 participants, predominantly (69.5%) from the western part of Saudi Arabia. Results showed that the participants were predominantly female (72.2%) and between 19 and 29 years old (51.5%). Table 1 shows additional details about the demographics of the sample and is based on the first six questions of the questionnaire.

### 3.2. Core question analysis

Table 2a shows the sources used to locate information about food safety and foodborne illnesses during crises; the Internet (*M* = 2.47) was the top-ranked source, followed by social media (*M* = 2.53).

**Table 2a.** Media preference matrix.

	Internet	TV	F2F	Newspaper	Phone	Social Media
Rank 1	103	33	13	17	27	102
Rank 2	63	43	35	43	45	66
Rank 3	57	53	38	56	46	45
Rank 4	39	64	55	45	47	45
Rank 5	24	70	66	52	59	24
Rank 6	9	32	88	82	71	13
**Average rank**	**2.47**	**3.65**	**4.32**	**4.08**	**3.95**	**2.53**

Note: * Average rank was computed as a weighted average. A lower average rank value indicates a higher level of preference.

Table 2b provides a preference comparison by demographic characteristics. Bold numbers represent p-values for comparisons between preference scores for each demographic variable and a source of information. For example, the p-value for gender and the Internet was p = 0.004, which indicates a statistically significant difference between males and females with respect to the preference of the Internet. However, in the vast majority of cases, there was no difference in social media preferences with respect to the examined socio-demographic characteristics.

Preferences for various types of social media were assessed; results showed that the top-ranked social media platforms used by participants were WhatsApp (*M* = 2.99), Snapchat (*M* = 3.72), YouTube (*M* = 4.08), Instagram (*M* = 4.46), and Facebook (*M* = 4.81) (Table 3). Numbers in Table 3 show how participants ranked the various social media platforms. For example, 35 participants gave a rank of 1 to Instagram, and 40 participants gave a rank of 2 to YouTube.

Participants were also asked about the various types of social media they currently use. Data showed that the highest penetration rates were for WhatsApp (79.5%), Twitter (71.5%), Snapchat (63.1%), YouTube (52.7%), and Instagram (44.1%). Most participants (79.5%) simultaneously used between two and four different social media sources.

Results also showed that participants often obtained information about food safety from the Saudi Food and Drug Authority (72.6%), Saudi Ministry of Health (55.4%), research studies (48.8%), personal physician (46.8%), family (37.7%), friends (35.3%), and the U.S. Food and Drug Administration (33.6%).

When responding to the types of information sought about food safety, responses varied. Most frequently, people looked for epidemiological information (52.5%), quantitative risk estimates (23.1%), types of foodborne infection (15.3%), food poisoning outbreak incidents (7.8%), government rules and regulation related to food safety (7.5%), and food safety inspection results related to restaurants (3.7%). We found that participants to the survey preferred videos (67.5%), articles (57.6%), infographics (55.3%), over simple pictures (7.8%), and cartoons (3.7%).

**Table 2b.** Comparison of preferred sources of information for finding information about food safety and foodborne illnesses during crises by various demographic characteristics.

		Internet		TV		F2F		Newspaper		Phone		Social Media	
		M	SD	M	SD	M	SD	M	SD	M	SD	M	SD
Marital Status	Married (78)	2.7	1.5	3.6	1.5	4.2	1.6	4.1	1.7	4.1	1.6	2.4	1.5
	Unmarried (202)	2.4	1.4	3.7	1.5	4.4	1.5	4.1	1.6	3.9	1.6	2.6	1.5
	Separated/Widowed/Divorced (15)	3.0	1.3	3.5	1.7	3.9	1.8	3.9	1.6	4.0	2.0	2.7	1.7
	**p-Value a**	**0.057**		**0.913**		**0.460**		**0.842**		**0.720**		**0.607**	
Age	Under 18 (40)	2.4	1.3	3.6	1.7	4.5	1.4	4.2	1.5	3.8	1.8	2.6	1.4
	19–29 (152)	2.5	1.5	3.7	1.5	4.2	1.6	4.1	1.6	4.0	1.6	2.5	1.5
	30–39 (60)	2.6	1.4	3.6	1.5	4.5	1.6	3.9	1.8	4.1	1.5	2.3	1.4
	40–49 (30)	2.5	1.6	3.4	1.4	4.6	1.3	4.1	1.5	3.9	1.8	2.5	1.6
	50 and over (13)	2.1	1.3	4.1	1.6	4.0	1.5	3.9	1.5	3.7	1.9	3.2	1.8
	**p-Value a**	**0.789**		**0.615**		**0.574**		**0.957**		**0.950**		**0.489**	
Region	Middle (23)	2.4	1.4	3.4	1.5	4.3	1.4	4.4	1.6	4.1	1.6	2.4	1.6
	Northern (10)	2.1	1.4	4.2	1.4	5.0	1.5	4.1	1.3	3.2	1.6	2.4	1.4
	Southern (33)	2.5	1.3	3.3	1.6	3.9	1.5	4.3	1.5	4.6	1.4	2.5	1.6
	Eastern (24)	2.5	1.2	4.0	1.7	4.8	1.4	3.6	1.8	3.7	1.8	2.4	1.2
	Western (205)	2.5	1.5	3.7	1.5	4.3	1.5	4.1	1.6	3.9	1.7	2.6	1.5
	**p-Value a**	**0.920**		**0.251**		**0.084**		**0.492**		**0.074**		**0.950**	
Gender	Female (213)	2.6	1.4	3.6	1.5	4.3	1.6	4.2	1.6	3.9	1.7	2.5	1.5
	Male (82)	2.1	1.5	3.9	1.5	4.4	1.4	3.9	1.7	4.2	1.5	2.5	1.4
	**p-Value b**	**0.004**		**0.114**		**0.636**		**0.187**		**0.242**		**0.599**	
Education	High school (23)	2.7	1.7	3.8	1.4	4.5	1.4	4.2	1.8	3.5	1.6	2.3	1.4
	Some college (140)	2.5	1.5	3.6	1.6	4.2	1.5	4.0	1.6	4.0	1.7	2.7	1.6
	Bachelor's (111)	2.4	1.4	3.7	1.6	4.4	1.6	4.2	1.6	4.0	1.5	2.4	1.4
	Master's (14)	1.3	0.6	4.0	0.0	5.7	0.6	2.7	0.6	4.0	2.6	3.3	1.5
	Other (7)	2.7	1.5	3.8	1.3	4.2	1.6	4.4	1.5	3.8	1.9	2.1	1.4
	**p-Value a**	**0.923**		**0.774**		**0.338**		**0.688**		**0.653**		**0.327**	

Note: ^a^ Kruskal Wallis H-test, b Mann Whitney U-test. * Lower mean rank represents a greater preference for a particular source.

**Table 3.** Preferred social media platforms for looking for information about food safety and foodborne illnesses during crises.

	Facebook	YouTube	Instagram	Twitter	LinkedIn	Snapchat	WhatsApp	Other
Rank 1	20	45	35	20	10	62	96	7
Rank 2	33	40	36	30	32	52	57	15
Rank 3	38	42	48	33	42	34	32	26
Rank 4	36	37	37	39	39	34	36	37
Rank 5	51	43	34	28	33	40	31	35
Rank 6	37	43	29	47	42	28	25	44
Rank 7	43	29	34	54	42	35	16	42
Rank 8	37	16	42	44	55	10	2	89
Average rank *	**4.81**	**4.08**	**4.46**	**5.04**	**5.11**	**3.72**	**2.99**	**5.79**

Note: * Average rank was computed as a weighted average. A lower average rank value indicates a higher level of preference.

The participants were also able to articulate their preferences regarding the characteristics of social media platforms when seeking information on foodborne illnesses and food safety (Table 4). Results showed that searchability (3.8 ± 0.93), presentation. and additional features (4.3 ± 0.75), familiarity (3.7 ± 0.98), timeliness (4.2 ± 0.85), and trustworthiness (3.7 ± 0.98) were the most important factors of consideration. An ANOVA test [22] indicated that there was a statistically significant difference between each dimension (*p* < 0.01).

Participants also rated the overall food safety conditions in the country as satisfactory (3.6 ± 0.54), and that food safety regulations in Saudi Arabia were adequate for meeting threats to food safety (3.9 ± 0.89).

**Table 4.** Top-ranked criteria when seeking information on food safety and foodborne illnesses over social media platforms.

Properties of Social Media Platforms	Mean	SD
Accuracy	2.2	0.92
Timeliness	4.2	0.85
Searchability (e.g., search function)	3.8	0.93
Security	2.1	1.01
Trustworthiness of the platform	3.7	0.98
Interactivity	2.5	1.01
Enhanced usability—visuals (e.g., pictures and videos)	4.3	0.75
Familiarity	3.7	0.98
**Question 14**		
How would you rate the overall food safety conditions of the country?	3.6	0.54
**Question 15**		
How confident are you that Saudi Arabian laws and regulations are effective in protecting the public against foodborne illnesses during crises?	3.9	0.89

## 4. Discussion

It is important to note that the data collected came predominantly from students and therefore may differ from those obtained from older adults. In general, the younger population uses social media to a greater extent than older population does. Online social media platforms appear to be their top choice as a mean to communicate, surpassing television and face-to-face interactions when seeking information, and get more knowledgeable about food safety [23]. This type of finding is not entirely unexpected, as digital media enables individuals to receive food safety information within seconds, a major advantage particularly in the midst of a major health crisis such as the COVID-19 pandemic [23]. The responses to questions 8 and 9 largely mirror each other, as it turns out that the social media preferences (Question 8) roughly correspond to the social media platforms used by the participants (Question 9). Such a correlation between the responses is reasonable because individuals who prefer a particular platform are also likely to use it, and vice-versa. Survey participants seemed to prefer the social media platform WhatsApp, but there were many other platforms also frequently used, such as Twitter and Snapchat. Thus, it is almost certain that reaching broad audiences to disseminate information about food safety and foodborne illnesses during crises can be better accomplished through multiple platforms rather than one only [15],[24],[25].

Social media preferences found in this study differ from those identified by other researchers. For example, a previous study by Ma et al. [26] quite similar to the one presented here, led to very different results, specifically to their popularity. For instant, in this study survey, Instagram was preferred over Facebook, whereas in the study by Ma et al. (2017), the opposite was found [26]. However, Ma et al. [26] study was found in agreement with respect to the ranking of sources of information to become more knowledgeable about food safety, such as the Internet, television, face-to-face discussion, printed materials, and phone, that participants preferred to access for information on food safety.

Another study conducted in the United Arab Emirates (UAE) reported on the distribution of social media in the (UAE) [27], and its results are close to those reported in the present study. For instance, in the UAE study, 31% of participants used Facebook; in our study, the proportion was 36.9% [27]. Similarly, 44.1% of this study used Instagram, but the value reported by Fathelrahman and Basarir was 36% [27]. Notably, 20% of the UAE study participants used Twitter, a quite different result from the 71.7% found in this study [27]. Unlike for Facebook and Instagram, for an unknown reason, the difference in the use of Twitter between the two studies appeared very different.

Saudi public health agencies such as the Food and Drug Authority and Ministry of Health were found in the current study to be the most trusted and frequently used sources of information about food safety. This finding suggests that there is an opportunity for these two agencies to expand their reach through the use of social media channels. The results presented here also provide some directions with respect to the type of information that should be provided and how this information should be delivered. These findings are consistent with those obtained by Jacob, Mathiasen, Powell [16] and Chapman et al. [23], also found that consumers may be interested in learning about the epidemiology of foodborne infections, through both visual and textual means.

The participants rated the food safety conditions and adequacy of food safety regulations in Saudi Arabia at 3.6 ± 0.34 and 3.9 ± 0.45, respectively. Considering that the maximum possible rating is five, these findings raise the question as to why no participants gave all five points. One possible explanation is that the participants were not very knowledgeable about the country's actual food safety conditions and/or were unfamiliar with its food safety regulations. In the future, more extensive studies may answer this question.

To the best of our knowledge, the results presented here are the first to highlight how individuals in Saudi Arabia use social media to acquire foodborne illnesses information and food safety knowledge during major health crises. Many of the results also appear to be in agreement with other previous studies performed by other independent research groups [26],[27]

## 5. Conclusions and outlook

The cross-sectional study presented here, although limited in scope and size, clearly demonstrates the importance of social media, in Saudi Arabia, as a mean to access information on food safety, and to get more knowledgeable about safe food handling, preparation, storage and hygiene in general. It was found that WhatsApp, Snapchat, and YouTube were the participants' preferred social media outlet and that the most trusted sources of information on food safety were the Saudi Food and Drug Authority (SFDA), and the Saudi Ministry of Health.

We are confident that the findings of this study will provide directions to Saudi policymakers, food safety public information campaigns, and Saudi public health agencies with respect to using social media to improve public awareness about food safety in a timely, fast, and efficient manner. Better knowledge will ultimately lead to improved food safety practices and standards in the country.

We also hope that our survey will serve as a precursor for more extensive future research studies that may explore the relationship between demographic characteristics and media preferences, and the effectiveness of various types of food safety messages. It would be interesting to expand the survey to a larger geographical area of Saudi Arabia, ultimately a large, nation-wide survey among individuals from various age groups and profiles.
