# Supplementary material for: Use of social media in food safety in Saudi Arabia—a preliminary study
Source: AIMS Public Health. 2021 Mar 29;8(2):322–32. doi: 10.3934/publichealth.2021025 (PMC8116190; doi:10.3934/publichealth.2021025)
Supplement: Supplementary file 1 [file publichealth-08-02-025-s001.pdf]

Public Health

AIMS Public Health, 8(2): 322–332.
DOI: 10.3934/publichealth.2021025
Received: 07 December 2020
Accepted: 23 March 2021
Published: 29 March 2021

http://www.aimspress.com/journal/aimsph

Research article

Use of social media in food safety in Saudi Arabia—a preliminary study

Nisreen M Abdulsalam[1,*] and Marwan A Bakarman[2]

[1] Department of Food and Nutrition, Faculty of Human Sciences and design, King Abdul Aziz University, P.O. Box 42807, Jeddah 21551, Saudi Arabia
[2] Department of Family and Community Medicine, Rabigh Faculty of Medicine, King Abdul Aziz University, P.O. Box. 80205, Jeddah 21589, Saudi Arabia

* **Correspondence:** Email: nabdulsalam@kau.edu.sa; Tel: +966567796333; Fax: +966126952005.

Supplementary

Please answer the following questions:

1. What is your gender?
   - ☐ Female
   - ☐ Male

2. How old are you?
   - ☐ Under 18
   - ☐ 19-29
   - ☐ 30-39
   - ☐ 40-49
   - ☐ 50 and over

3. What is your marital status?
   - ☐ Married
   - ☐ Divorced
   - ☐ Separated
   - ☐ Widowed
   - ☐ Unmarried

4. What is your current employment status?
   - ☐ Full-time employment
   - ☐ Part-time employment
   - ☐ Unemployed
   - ☐ Self-employed
   - ☐ Home-maker
   - ☐ Student
   - ☐ Retired
   - ☐ Other______________________

5. What is your highest level of education?
   - ☐ High school
   - ☐ Some college
   - ☐ Bachelor’s degree
   - ☐ Master’s degree
   - ☐ Doctoral degree
   - ☐ Professional degree
   - ☐ Other______________________

6. Region of country
   - ☐ Middle Region
   - ☐ Northern Region
   - ☐ Southern Region
   - ☐ Eastern Region
   - ☐ Western Region

7. Rank sources of information that you will use to locate information about foodborne illnesses and food safety during crises? (Arrange in a preferred order, 1 is the most preferred, 6 is the least preferred)

- ☐ Internet search
- ☐ TV
- ☐ Face to face
- ☐ Newspaper, book, magazine, or other printed material
- ☐ Phone call or text messages
- ☐ Social media platform

8. Rank social media platforms will you use to look for information about foodborne illnesses and food safety during crises? (Arrange in a preferred order, 1 is the most preferred, 6 is the least preferred)

- ☐ Facebook
- ☐ YouTube
- ☐ Instagram
- ☐ Twitter
- ☐ LinkedIn
- ☐ Snapchat
- ☐ WhatsApp
- ☐ Other____________

9. Which social media platforms do you currently use? (check all that apply)

- ☐ Facebook
- ☐ YouTube
- ☐ Instagram
- ☐ Twitter
- ☐ LinkedIn
- ☐ Snapchat
- ☐ WhatsApp
- ☐ Other____________

10. Which of the following sources do you use to get information about food safety? (check all that apply)

- ☐ Saudi Ministry of Health
- ☐ Saudi foods and drug authority
- ☐ Personal physician
- ☐ Friends
- ☐ Family
- ☐ From foreign food safety agencies, such as U.S. Food and drugs administration
- ☐ Research studies
- ☐ Other______________

11. What kind of food safety information you are looking for when you use social media platforms? (check all that apply)?

- ☐ Recalls
- ☐ Food poisoning outbreak incidents
- ☐ foodborne illness in general
- ☐ Food safety information in general
- ☐ Food safety inspections results related to restaurants
- ☐ Safe food handling
- ☐ Government rules and regulation related to food safety
- ☐ Food fraud/adulteration
- ☐ Epidemiological information
- ☐ Quantitative risk estimates
- ☐ Types of foodborne infections
- ☐ Information on how microorganisms cause foodborne illnesses
- ☐ Food labeling issue
- ☐ Food expiry date information
- ☐ Dietary supplements information
- ☐ Other____________

12. How would like the information to be presented in your favorite social media platforms? (check all that apply)?

- ☐ infographic
- ☐ video
- ☐ article/text
- ☐ picture
- ☐ cartoon
- ☐ podcast
- ☐ snippet
- ☐ interview with experts
- ☐ Q & A
- ☐ Other____________

13. Which properties of social media platforms are most important for you when looking for information about foodborne illnesses and food safety during crises? (Please specify your preferences)

	**Unimportant**				**Very important**
Accuracy	1	2	3	4	5
Timeliness	1	2	3	4	5
Searchability (e.g., search function)	1	2	3	4	5
Security	1	2	3	4	5
Trustworthiness of the platform	1	2	3	4	5
Interactivity	1	2	3	4	5
Enhanced usability – visuals (e.g., pictures and videos)	1	2	3	4	5
Familiarity	1	2	3	4	5

14.

	**Extremely bad**				**Extremely good**
[illegible]ow would you rate the overall food safety condition [illegible]f the country?	1	2	3	4	5

[illegible]5.

	**Not confident at all**				**Very confident**
[illegible]ow confident are you that Saudi Arabian laws and [illegible]egulations are effective in protecting the public [illegible]gainst foodborne illnesses during crises?	1	2	3	4	5

16. In your view, what is the best method to use social media for disseminating and/or receiving information about foodborne illnesses and food safety? (Please type your response)

____

____

Thank you for participating!

AIMS Press

© 2021 the Author(s), licensee AIMS Press. This is an open access article distributed under the terms of the Creative Commons Attribution License (http://creativecommons.org/licenses/by/4.0)
